# Is It Time to Reassess the Role of Radiotherapy Treatment in Ovarian Cancer?

**DOI:** 10.3390/healthcare11172413

**Published:** 2023-08-28

**Authors:** Gabriella Macchia, Francesca Titone, Stefano Restaino, Martina Arcieri, Giulia Pellecchia, Claudia Andreetta, Lorenza Driul, Giuseppe Vizzielli, Donato Pezzulla

**Affiliations:** 1 Radiation Oncology Unit, Responsible Research Hospital, 86100 Campobasso, Italy; macchiagabriella@gmail.com (G.M.); pezzulla.donato@libero.it (D.P.); 2 Radiation Oncology Unit, Department of Oncology, “Santa Maria della Misericordia” University Hospital, Azienda Sanitaria Universitaria Friuli Centrale (ASUFC), 33100 Udine, Italy; 3 Department of Maternal and Child Health, “Santa Maria della Misericordia” University Hospital, Azienda Sanitaria Universitaria Friuli Centrale (ASUFC), 33100 Udine, Italy; restaino.stefano@gmail.com (S.R.); martina.arcieri@unime.it (M.A.); giulia.pellecchia95@gmail.com (G.P.); lorenza.driul@uniud.it (L.D.); giuseppe.vizzielli@uniud.it (G.V.); 4 Department of Biomedical, Dental, Morphological and Functional Imaging Science, University of Messina, 98122 Messina, Italy; 5 Medical Area Department (DAME), University of Udine, 33100 Udine, Italy; 6 Medical Oncology Unit, Department of Oncology, “Santa Maria della Misericordia” University Hospital, Azienda Sanitaria Universitaria Friuli Centrale (ASUFC), 33100 Udine, Italy; claudia.andreetta@asufc.sanita.fvg.it

**Keywords:** ovarian cancer, radiation therapy, radiotherapy targeted

## Abstract

With a 5-year survival rate of fewer than 50%, epithelial ovarian carcinoma is the most fatal of the gynecologic cancers. Each year, an estimated 22,000 women are diagnosed with the condition, with 14,000 dying as a result, in the United States. Over the last decade, the advent of molecular and genetic data has enhanced our understanding of the heterogeneity of ovarian cancer. More than 80% of women diagnosed with advanced illness have an initial full response to rigorous therapy at diagnosis, including surgery and platinum-based chemotherapy. Unfortunately, these responses are infrequently lasting, and the majority of women with ovarian cancer suffer recurrent disease, which is often incurable, despite the possibility of future response and months of survival. And what therapeutic weapons do we have to counter it? For many years, radiation therapy for ovarian tumors was disregarded as an effective treatment option due to its toxicity and lack of survival benefits. Chemotherapy is widely used following surgery, and it has nearly completely supplanted radiation therapy. Even with the use of more modern and efficient chemotherapy regimens, ovarian cancer failures still happen. After receiving first-line ovarian cancer chemotherapy, over 70% of patients show evidence of recurrence in the abdomen or pelvis. It is necessary to reinterpret the function of radiation therapy in light of recent technological developments, the sophistication of radiation procedures, and the molecular and biological understanding of various histological subtypes. This review article focuses on the literature on the use of radiation in ovarian tumors as well as its rationale and current indications.

## 1. Introduction

Ovarian cancer (OC) is the most severe gynecologic malignancy. Each year, an estimated 22,000 women are diagnosed with the condition, with 14,000 dying as a result in the United States [1]. Since the typical pattern of spread is malignant dispersion throughout the peritoneal cavity prior to the onset of any symptoms, in reality most women present with advanced-stage disease. The International Agency for Research on Cancer (IARC) GLOBOSCAN 2018 highlights the need for better and more effective treatments for OC, especially in younger women, who represent the majority of cases (78% cases < 70 years) and have a 5-year death rate of over 50% [1,2]. Despite the fact that more than 80% of advanced disease patients have an initial complete response after an aggressive treatment strategy, these responses are infrequently durable, generally with a subsequent recurrence development. Many trials have been conducted in recent years to examine potential drugs to improve treatment for advanced OC patients with the goal of extending survival. Poly (ADP-ribose) polymerases inhibitors (PARPi) are a potential treatment option studied in many trials. To date, the greatest impact was seen in the maintenance setting, where it was shown to extend the progression-free survival of OC patients, particularly those with a BRCA1/2 mutation [3]. Unfortunately, subsequent chemotherapeutic regimens were characterized by short responses due to the progressive development of resistance mechanisms, leading to the need for new therapeutic approaches.

One of these potential strategies could be radiotherapy, especially given OC’s documented radiosensitivity. Consolidative irradiation of the peritoneal cavity was the cornerstone of adjuvant therapy in this context to sterilize micrometastatic disease for many years, before being replaced by cisplatin roughly three decades ago. As a result of its severe side effects and low therapy compliance rates, it was removed from conventional treatment, remaining a viable but underutilized therapeutic alternative for patients with chemoresistance or refractory disease [4]. Nowadays, radiotherapy is receiving more and more attention due to the development of new techniques with lower toxicity rates, such as Intensity Modulated Radiotherapy (IMRT) and Volumetric Modulated Arc Therapy (VMAT), as well as the potential for synergistic interactions with new pharmaceutical agents, such as PARPi. In truth, radiotherapy was regarded as an effective therapy for women with peritoneal micrometastatic disease following surgery, focal metastatic disease, ovarian clear cell carcinoma, and palliation of advanced disease. Radiotherapy could be reconsidered as part of the standard management for this deadly disease through newer radiotherapy techniques such as stereotactic body radiotherapy (SBRT) and low-dose hyperfractionation in combination with targeted agents [5]. The purpose of this narrative review is to describe in detail the different therapeutic possibilities as well as the evolution of the radiation strategy during the past few decades in the context of OC, particularly in light of the new opportunities emerging from a technical and pharmacologic perspective.

## 2. Relevant Sections

### 2.1. Whole Abdomen Radiotherapy (WART)

In approximately 85% of patients, OC is characterized by a transperitoneal dissemination pattern into the abdominal and pelvic cavities. Whole abdomen radiotherapy (WART) is an irradiation technique that involves the entire abdominal area being treated by conventional photon therapy. It is often used to treat cancers that have spread to the abdominal area, such as OC or lymphoma, or to prevent the spread of cancer cells from the abdominal area to other parts of the body. WART was proposed as a pre-chemotherapy treatment with the aim of sterilizing large volumes of micrometastatic intraperitoneal seeding, overcoming the limits of partial irradiation [5]. Sorbe et al. [6], in a large prospective randomized trial, describe how WART might play an important role in this setting. The authors described 98 advanced OC patients with complete pathologic remission following adjuvant chemotherapy, demonstrating a benefit in terms of recurrence rates, progression free survival (PFS), and overall survival (OS) compared with chemotherapy or no further treatment. Abdominal radiotherapy (1.0 Gy per fraction, 5 days a week, 20 fractions, and 12–18 MV energy) was given via open Antero-Posterior fields from the domes of the diaphragm to the pelvic floor without shielding. Another 20.4 Gy (1.7 Gy per fraction, 5 days a week, and 12 fractions) were given as a boost dose via the lower (L3–4-disc level to the pelvic floor) abdominopelvic Antero-Posterior fields. The radiotherapy group experienced greater treatment-related side effects, particularly late intestinal issues. This study represents one of the best pieces of evidence supporting WART, but several smaller studies and a systematic review found that radiation therapy following radical surgery improves disease-free survival in patients with advanced-stage OC [7,8,9,10,11]. However, the oldest series were characterized by a high toxicity profile in terms of fatigue, nausea, and diarrhea as well as a poor therapeutic compliance rate [6,8,9,12,13,14,15], forcing the discontinuation of this method. Since OC is rarely restricted to the pelvis, whole pelvic radiation was deemed largely ineffective for disease control since it does not treat the entire volume at risk of recurrence. The low doses required to achieve bowel, kidney, and liver tolerance utilizing two-dimensional fields were ineffective in eradicating the macroscopic residual disease in the peritoneal cavity, resulting in poor treatment efficacy. Furthermore, the toxicity of radiation therapy was significant, especially when applying wide-field irradiation. Nowadays, WART is being revisited as a feasible treatment method due to technical improvements in the radiotherapy arena, with the implementation of techniques like IMRT or VMAT, which allow for exceedingly precise irradiation of complex target volumes while preserving the normal tissues in the surrounding areas.

A first step in this direction was represented by the OVAR-IMRT-01 trial, a phase I study that investigated the use of Intensity-Modulated Radiotherapy (IMRT) as a consolidation therapy after adjuvant carboplatin/taxane chemotherapy. In the treatment of complex cancers that are adjacent to organs at risk, IMRT has supplanted three-dimensional conformal radiotherapy. It modulates and shapes the beam with static or dynamic beams using a computer algorithm to optimize the dose to the target while minimizing exposure to at-risk organs. This breakthrough trial enrolled 10 patients, administering IMRT WART up to a total dose of 30 Gy in 1.5-Gy fractions. The results showed that all patients completed the treatment without any toxicity-related interruptions, with minimal side effects, including four cases of Grade 3 toxicities (one each of diarrhea, thrombocytopenia, and leukopenia) [7]. These encouraging findings prompted the OVAR-IMRT-02 study, a multicenter single-arm phase II trial that showed a tolerable risk of acute and late toxicity and had a negligible influence on the long-term quality of life in 20 OC patients treated in the entire peritoneal cavity with 30 Gy in 20 fractions of IMRT WART [16]. In terms of late toxicity, no grade 4 adverse event was recorded, and the most common were grade 1 and 2 lower limb edemas (44.5%). Furthermore, the trial revealed intriguing data on PFS and OS, with estimated 1-, 2-, and 3-year PFS of 74%, 51%, and 40% and 1-, 2-, and 3-year OS of 89%, 83%, and 83%, respectively [16]. For the sake of clarity, we recognized that in both OVAR-IMRT-01 and OVAR-IMRT-02, radiotherapy followed a complete remission after optimal cytoreduction (postoperative residual tumor of less than 1 cm) plus six courses of chemotherapy.

A major innovation in this setting was the development of an in-house automated dual iso-center volumetrically modulated arc-based therapy technique (VMAT). VMAT experiences are newer and, therefore, less represented, but they are just as effective. Mahantshetty et al. conducted a study that compared the dosimetric features of IMRT and VMAT in WART treatment. The results showed that VMAT provided encouraging dosimetric features with improved logistics over IMRT [17]. Furthermore, Stevens et al. conducted a study using a VMAT approach for WART treatment on a small sample of five patients [18]. The acute toxicities, which occurred 2–6 weeks after WART, were lower than Grade 3 and included low-grade persistent nausea, diarrhea, and cysto-urethritis. The main late toxicities were low-grade lymphopenia in four patients, minor elevations in liver function, and one case of severe (G3) gastrointestinal injury (enterocolitis was detected 9 months after WART, requiring surgical intervention). Furthermore, all patients were alive and progression-free at the clinical, biochemical (CA-125), and 18Fluoro-deoxyglucose (FDG) PET/CT re-evaluation after a median follow-up of 77 months (range 16–83) [18].

Based on these findings, we may conclude that IMRT WART can be considered as a potential therapy option for all patients with advanced-stage OC, characterized by great treatment tolerance, an acceptable toxicity profile, and just a modest impact on long-term quality of life. More randomized trials are needed to further analyze the OVAR-IMRT-02 trial’s impressive PFS and OS rates. Furthermore, the combination of WART and PARP inhibitors and bevacizumab should be investigated.

### 2.2. Low-Dose Fractionated Whole Abdominal Radiation (LDFWART)

The hypothesis of using low-dose radiotherapy during chemotherapy as a chemosensitizer represents a paradigm shift in respect to the use of low-dose chemotherapy during radiation treatment as a radiosensitizer. There was much increased apoptotic activity in cells treated with low-dose radiation, according to the literature [19,20]. Due to technological radiotherapy advancements and increased safety, new trials using low-dose fractionated whole abdominal irradiation (LDFWART) as a “chemopotentiator” factor, such as those presented by Kunos et al. [21] and Ngoi et al. [22], emerged. These trials aimed to investigate the potential benefits of combining chemotherapy with low-dose radiation therapy. Kunos et al. presented a phase I trial in which 13 patients affected by recurrent epithelial ovarian, fallopian tube, or peritoneal cancers were enrolled to identify the maximum tolerated dose and dose-limiting toxicity of LDFWART with weekly docetaxel as a chemosensitizer. They proposed a tolerable combination of twice-weekly LDFWART (60 cGy) and weekly docetaxel 20 mg/m^2^ with a median progression-free survival of 3.3 months [21]. On the basis of these promising results, Ngoi et al. then designed a phase I trial in a similar setting to identify the recommended phase 2 dose and preliminary activity of weekly paclitaxel concurrent with LDFWART in platinum-resistant OC patients. Ten patients were recruited, with neutropenia (60%) and anemia (30%) being the most common grade 3 adverse events, and 80 mg/m^2^ was the recommended phase 2 dose. Moreover, the median progression-free survival and overall survival were 3.2 and 13.5 months, respectively, with four patients having 12 weeks of disease control, after completing 12–21 weeks of weekly Paclitaxel. In conclusion, in this heavily pre-treated population, the authors reported encouraging efficacy and suggested that weekly Paclitaxel + LDFWART may be useful in the context of platinum-resistant OC, especially when bevacizumab is contraindicated [22]. Recently, the introduction of PARPi led to a further rediscovery of LDFWART. BRCA1/2 mutations are found in 15–25% of high-grade serous ovarian carcinomas (HGSOC). Poly (ADP-ribose) polymerase (PARP) inhibition is synthetically lethal to cells and tumors with BRCA1/2 mutations. Importantly, it was ovarian cancer that led to the discovery of the concept of “synthetic lethality” when cell lines with the homozygous deletion or inactivation of BRCA1/2 were treated with PARPi. The principle of “synthetic lethality” states that chemical agents that inhibit a specific pathway are synthetically lethal with a mutation or genetic lesion that prevents a salvage or alternative pathway that is essential for survival. Numerous studies and clinical trials involving PARPi in cancers with BRCA1/2 genetic abnormalities have corroborated similar findings since then. It is now well-known, in fact, that PARP enzymes are involved in base excision DNA repair; in particular, PARP-1 and PARP-2 localize the damaged DNA and catalyze the transfer and polymerization of Poly(ADP-ribose) [23,24,25,26,27]. PARP inhibition was particularly used in cancers with breast cancer (BRCA) genes’ mutations [28,29,30]. BRCA-mutant cancer cells are characterized by the abnormal Homologous Recombination (HR) repair of DNA. In these tumors, the Base Excision Repair (BER) pathway is important for cell survival. PARP enzymes play an essential role in BER, and PARPi are effective in causing cell death in BRCA-mutant cells while sparing normal cells (i.e., synthetic lethality). Indeed, PARPi are the first cancer therapy aimed at exploiting synthetic lethality [31]. However, even in the absence of these mutations, different experiences showed how PARPi may function as sensitizing agents for chemotherapy and radiotherapy [27,28,29,30,31]. According to these preclinical and clinical data, Reiss et al. proposed a phase I study aimed at verifying the safety and efficacy of LDFWAR plus PARPi in patients with peritoneal carcinomatosis, a setting with minimal therapeutic options [32]. Twenty-two patients with advanced solid tumor malignancies and peritoneal carcinomatosis (eight patients had primary ovarian or fallopian cancer) received veliparib for a total of three cycles (80–320 mg daily). LDFWAR consisted of 21.6 Gy in 36 fractions and 0.6 Gy twice daily on days 1 and 5 for weeks 1–3 of each cycle. LDFWAR was delivered using anterior and posterior open fields with posterior kidney shielding used to keep kidney doses under 20 Gy. Lymphopenia (68%), thrombocytopenia (14%), and anemia (9%), were the most common toxicities. In terms of PFS and OS, the median PFS was 4.47 months, and the median OS was 13.04 months. The median PFS and OS for the eight ovarian and fallopian cancer patients were 6.77 months and 17.54 months, respectively [32]. The encouraging results in the ovarian subset prompted a more focused trial in which LDFWAR was combined with veliparib in patients with peritoneal carcinomatosis, with dose escalation in patients with ovarian and fallopian cancer. Once again, the most common treatment-related grade 3 and 4 toxicities in 32 patients were lymphopenia (59%), thrombocytopenia (12%), and anemia (9%). The median PFS was 3.6 months, and the median OS was 9.1 months, with platinum-sensitive patients having a longer OS (10.9 months) than platinum-resistant patients (5.8 months). In conclusion, the authors found this strategy tolerable, despite the fact that fewer than half of the patients were able to finish the regimen, either due to disease progression or adverse events [33].

### 2.3. Stereotactic Body Radiotherapy (SBRT)

OC patients may also experience progressive disease under a completely different scenario, known as oligometastases, oligopersistence, or oligorecurrence [34], either in patients undergoing major surgery or in patients receiving multiple lines of chemotherapy. Indeed, oligometastatic disease is an intermediate state between localized and systemically metastasized disease with a limited number of metastases, between three and five in a number of sites ≤3, that is still not acquiring the potential for widespread metastases and that could benefit from localized metastases-direct ablative therapy such as surgical resection or radiosurgery. Stereotactic body radiotherapy (SBRT) is a minimally invasive technique, which allows for the precise delivery of converging high-dose radiation beams in a short time (one–five treatments), with a limited dose delivered to the surrounding normal tissue. SBRT offers high local control with minimal toxicities, can be safely administered between cycles of chemotherapy, and allows for retreatment in previously irradiated patients. Moreover, a very strong biological and radiobiological rationale is related to the high dose per fraction delivery because the massive cell killing results in antigen release through the cellular microenvironment, helping to set off the immune system’s response [35]. All the reported SBRT features can lead to an improvement in quality of life and delay the shift to other chemotherapy lines. Therefore, a plethora of clinical studies collected evidence on this technique. The phase II SABR-COMET trial, in particular, showed a higher median overall survival (OS) as well as higher toxicity in the SBRT arm. In this trial, 99 patients with a range of controlled primary tumors (lung, colorectal, breast, and prostate cancer) and one–five oligometastases were randomly assigned to standard palliative treatment with or without SBRT for all metastatic lesions [36]. Moreover, a 2020 worldwide survey found that the majority of radiation oncologists consider SBRT to be a standard rescue option for nodal and locally recurrent gynecologic diseases [37]. However, due to the lack of randomized evidence, the effectiveness of SBRT for oligometastatic gynecologic malignancies is still unknown. A recent review analyzing the role of SBRT in gynecologic oncology concluded that it is an active area of investigation, the literature supports the local control data in the setting of limited metastatic disease, and its safety in terms of toxicity is established by phase I and II trials, though there is a scarcity of phase III randomized controlled trials, including the use of immunotherapy [38]. Many studies have been conducted in recent years to investigate the role of SBRT in metastatic OC. Trippa et al. used SBRT (25–40 Gy in five daily fractions) in 11 patients, with only lymph nodes involved, with a median follow-up of 24 months, and the complete response (CR) was 100%; the 2-year local progression-free survival (PFS) was 73%, and the 2-year OS was 78% [39]. In a study by Iftode and colleagues, SBRT was used to treat 26 patients with 44 metastatic lesions. Lung metastases received 48 Gy in four fractions, liver metastases received a median total dose of 75 Gy in three fractions, and lymph node lesions received a median total dose of 45 Gy in six fractions. The two-year LPFS, two-year PFS, and two-year OS rates were 92.9%, 38%, and 92.7%, respectively, with a median follow-up of 28.5 months. The radiosensitivity of the endometrioid and clear-cell histology was demonstrated by this investigation [40]. Lazzari et al., in 82 patients with platinum-resistant OC, used SBRT (median of 24 Gy in three fractions) for the treatment of 156 metastatic lesions. The authors reported a 2-year local control (LC) of 68% and a median systemic treatment-free survival after SBRT of 7.4 months with no severe toxicities. This study demonstrated that OC is radiosensitive, even in the presence of platinum-resistant disease [41]. Macchia et al., in the largest multicentric retrospective study (the MITO RT1 study), included 261 patients (449 lesions). Age ≤ 60 years, volume ≤ 18 cc, and nodal disease treated with BEDα/β10 > 70 Gy were significantly associated with a higher probability of achieving a complete response (CR) and local control. With a median follow-up of 22 months, the 2-year LC rate and 2-year OS were 81.9% and 73.8%, respectively, with no severe toxicity. In this study, half of the patients were >60 years, 46% of patients received ≥2 previous lines of chemotherapy, and at least one major surgery was performed. Therefore, the authors concluded that SBRT has to be considered an option for treatment in oligometastatic OC due to its safety, even in an unfit setting [42]. In a 2018 study, Yue Bi et al. assessed the sensitivity of OC cells with a BRCA1 mutation to the PARP inhibitor in vitro. Their study showed how the “synthetic lethality” mechanism of PARP inhibitors radiosensitizes cells with the BRCA mutation, making them highly vulnerable to the effects of radiotherapy [43]. There are a few studies in the literature, particularly phase I trials, that examined the relationship between PARP inhibitors and radiation in various primary diseases (lung, colon–rectal, brain, and breast) [44,45,46,47], and only one of them used stereotactic fractionation; however, it was a mixed series, reporting surgery or radiotherapy as metastases-directed therapy [48]. In detail, Palluzzi et al. reported on the data of 30 OC patients with oligometastatic progression under PARPi maintenance therapy. Ten patients (33%) underwent local treatment with surgery, and twenty patients (67%) underwent local treatment with stereotactic radiotherapy. Treatment with PARP inhibitors was continued until there was further disease progression. The median treatment-free interval among these patients increased by 9 months (95% CI 5.8 to 12.16) as a result of this tumor-directed strategy, significantly postponing the start of additional cytotoxic treatment. SBRT was the strategy of choice for the majority of patients; however, both approaches had few serious complications and no differences in terms of survival benefit [48]. Lastly, the retrospective multicenter Epimetheo study, to date still only reported in abstract form, investigated the activity and safety of SBRT in association with PARPi in oligometastatic OC patients. SBRT was used to treat 57 OC patients with a total of 115 lesions under PARPi maintenance. The one-year actuarial local control rate in patients achieving complete response was 94.2%, with encouraging toxicity data [49].

### 2.4. Magnetic Resonance Linear Accelerator (MRI-Linac) Radiotherapy

The Magnetic Resonance Linear Accelerator (MRI-Linac) is a novel and rapidly evolving technology with an importance that has grown exponentially in recent years. This method enables online adaptive external beam radiation based on considerable soft tissue contrast [50,51], obtaining a smaller planning target volume margin, better sparing of the organs at risk, dose escalation, and hypofractionation [52,53]. Even mobile targets can be treated with amazing precision and be tailored to the patient’s evolving anatomy, especially when the tumor and surrounding healthy tissues are directly visible. This could boost the dose delivered to the lesion while reducing the risk of side effects. Due to all of these contributing factors, this strategy offers a compelling therapeutic alternative for OC patients with pelvic and/or abdominal lesions. As the majority of these patients have lesions in this region, and the volumes of interest are typically found around the bowel area, it might be challenging to prescribe high dosages without also increasing the risk of toxicity. To give greater doses to target volumes while sparing adjacent tissues, organ motion has to be monitored during therapy, which MRI-Linac makes possible [54,55]. Werensteijn-Honingh et al. [52] were one of the first authors to describe the use of MRI-Linac in this clinical setting: they presented a feasibility study of SBRT using a 1.5 Tesla MRI-Linac for pelvic lymph node oligometastases in five patients, with excellent clinical and dosimetric results. In contrast, Yavas et al. [55] described the more practical clinical experience in this setting. They reported the cases of two OC patients with recurrent lesions in the parailiac region, treated with high field 1.5-Tesla MRI-Linac treatment in five fractions at a dose of 30 Gy. After 12 and 20 months of MRI-Linac radiotherapy, the patients were disease-free, with no significant toxicity. Recently, Henke et al. [56] reported the results of a phase I trial investigating the feasibility and safety of Stereotactic MRI-Guided Online Adaptive Radiation Therapy (SMART) in 10 patients affected by oligometastatic OC accounting for 17 lesions: the target was mainly the pelvic and abdominal regions, and only a severe acute toxicity (duodenal ulcer) was reported. Moreover, the local control at 3 months was 94%, with a median progression-free survival of 10.9 months.

A summary table of all the mentioned radiotherapies with their pros and cons can be found in the Conclusions section.

## 3. Conclusions

Even though OC is a known radiation-sensitive disease, radiation therapy did not historically gain popularity in this setting, especially due to the toxicity profile linked to the technological limits of the techniques used until then. However, the updated molecular knowledge, coupled with the technological radiation oncology advances in radiotherapy for OC patients, has regained appeal. To make it easier for readers to compare, a summary table of each mentioned radiation treatment, together with its benefits and drawbacks, is provided (Table 1). More robust clinical experience will be able to confirm whether the expected gain in these cases will translate into improved clinical outcomes. 

## 4. Future Directions

Immunotherapy has transformed cancer treatment in recent years. The immunological environment is critical to ovarian cancer prognosis, and efforts are being made to translate this into new therapeutic applications. More information on the combination’s role in this setting will be provided by a phase 2 trial of a PARP inhibitor with or without radiation in ovarian cancer. Radiation can induce multiple forms of DNA damage. The presence of defective non-homologous end joining (NHEJ) was demonstrated in ovarian cancer cell lines, and its inhibition led to persistent DNA damage, which in turn made cells more radiation-sensitive. An enhanced radiation response with inhibitors of NHEJ was demonstrated in other kinds of cancer cell lines and may be rational in ovarian cancer as well [5]. A further emerging scenario is represented by the possible interplay between the tumor microenvironment and radiotherapy: the radiation treatment could exert a great influence on it, which, in turn, could significantly affect the response to radiotherapy [57].

## Figures and Tables

**Table 1:** Benefits and drawbacks of each mentioned Radiotherapy technique/facility.

Radiotherapy Technique/Facility	Pros	Cons
Whole abdomen radiotherapy (WART)	Acceptable toxicity profile with new radiotherapy techniques Great treatment tolerance Potential therapy option for all patients with advanced-stage disease	Modest impact on long-term quality of life
Low-dose fractionated whole abdominal radiation (LDFWART)	Chemosensitizer with the ability to enhance the action of various drugs without the toxic effects of full-dose radiotherapy Tolerable strategy	Few studies on unselected populations
Stereotactic body radiotherapy (SBRT)	Minimally invasive Precise delivery of converging high-dose radiation beams in short time (one–five treatments) Limited dose delivered to the surrounding normal tissue High local control rates Minimal toxicities Safe administration between cycles of chemotherapy Retreatment allowed Set off the immune system’s response, because a high dose per fraction delivery causes a massive cell killing resulting in antigens’ release through the cellular microenvironment Advantageous cost-effective ratio	Importance of the patients’ selection
Magnetic Resonance Linear Accelerator (MRI-Linac)	Online plan adaptation Very small planning target volume margin High organs at risk sparing Mobile targets treatments Organ motion monitoring during therapy	Importance of the patients’ selection Expensive facility Lack of Magnetic Resonance capacity in radiotherapy departments
